# Anti-Inflammatory Effects of Zinc Oxide and Berberine in Rats with Dextran Sulfate Sodium (DSS)-Induced Colitis

**DOI:** 10.3390/ani14131919

**Published:** 2024-06-28

**Authors:** Seon-Hyoung Kim, Rangyeon Lee, Jang-Won Yoon, Hee-Tae Cheong, Chang-Six Ra, Ki-Jong Rhee, Jeongho Park, Bae-Dong Jung

**Affiliations:** 1 College of Veterinary Medicine, Kangwon National University, Chuncheon 24341, Republic of Korea; 2 Multidimensional Genomics Research Center, Kangwon National University, Chuncheon 24341, Republic of Korea; 3 Institute of Veterinary Science, Kangwon National University, Chuncheon 24341, Republic of Korea; 4 College of Animal Life Sciences, Kangwon National University, Chuncheon 24341, Republic of Korea; 5 Department of Biomedical Laboratory Science, College of Software and Digital Healthcare Convergence, Yonsei University Mirae Campus, Wonju 26493, Republic of Korea

**Keywords:** DSS-induced colitis, ZnO, berberine, swine, inflammation

## Abstract

**Simple Summary:**

Zinc oxide (ZnO) can be used to prevent intestinal diseases in the swine industry but its secretion in feces can result in environmental pollution. As an alternative for ZnO, we evaluated the action of berberine in rats with dextran sulfate sodium (DSS)-induced colitis. Berberine is an isoquinoline alkaloid known for regulatory action for inflammation. We compared ZnO with berberine and observed a protective effect of berberine to a similar level of ZnO. Our study suggests that berberine is a valid substitute for ZnO that could reduce intestinal inflammation and environmental pollution.

**Abstract:**

Zinc oxide (ZnO) is frequently used in high concentrations to prevent diarrhea in weaning pigs. However, it can produce environmental pollution, because it is not absorbed by the intestines and is excreted in the feces. In studies to identify an alternative substance to ZnO, we used a model of colitis induced by dextran sulfate sodium (DSS) in rats to compare the anti-inflammatory effects of berberine with ZnO. DSS-treated rats displayed weight loss, shortening of the colon, increased fecal water content, and an increase in the disease activity index (DAI). In contrast, DSS + ZnO- and DSS + berberine-treated rats exhibited reduced colon shortening, decreased fecal water content, and a decrease in the DAI. Histological analysis revealed that both ZnO and berberine treatment reduced epithelial cell damage, crypt destruction, and infiltration of inflammatory cells. Moreover, the liver damage index was not significantly different between ZnO and berberine-treated rats. This study indicated that both ZnO and berberine can improve DSS-induced colitis in rats and suggests berberine as an alternative treatment to ZnO that would not cause environmental pollution.

## 1. Introduction

Zinc (Zn) is an essential trace element that is involved in numerous enzymatic processes. It is also important for growth, reproduction, immune function, and the development of the digestive system [1,2,3,4,5,6]. A pharmacological dose of zinc oxide (ZnO) (2000–3000 mg/kg) promotes growth, positively modulates gut microbiota, and reduces the incidence of diarrhea in piglets [1,7,8,9]. Therefore, ZnO is commonly used in the swine industry, including its addition to piglet feed [1,2,8]. However, excessive ZnO that is not utilized is excreted in the feces, leading to environmental pollution, such as soil and water contamination [5,10]. Therefore, the supplementation of piglet diets with concentrated ZnO is restricted [2]. For example, ZnO usage for the prevention of diarrhea has been banned in the EU from 2022 [6]. Nevertheless, many countries, including Korea, still use 2000 ppm of ZnO in piglet feed. 

Berberine is an isoquinoline alkaloid known for its anti-inflammatory action, inhibition of DNA and protein synthesis, suppression of cell cycle progression, and anti-cancer effects [11,12,13,14,15]. Berberine has minimal side effects and resistance, and it directly exhibits antibacterial effects against pathogenic bacteria in the gut [16]. It also has a protective effect against damage caused by lipopolysaccharides (LPS), a major component of the outer membrane of Gram-negative bacteria [14,17,18,19,20] and is used for the treatment of bacterial diarrhea [13,15,21].

Major clinical symptoms of ulcerative colitis are diarrhea, bloody stools, weight loss, and anemia [22]. These symptoms are also observed in post-weaning diarrhea (PWD) in pigs [9]. Therefore, to investigate the potential of berberine for use as a substitute for ZnO in the swine industry, we compared the ability of berberine and ZnO treatment to prevent symptoms in rats with a DSS-induced colitis model. Notably, berberine treatment reduced both clinical symptoms and the inflammatory index during colitis to a similar degree to ZnO. Furthermore, hepatic enzyme levels were well controlled by berberine treatment. Our study suggests that berberine is a valid substitute for ZnO that could reduce intestinal inflammation and environmental pollution. 

## 2. Materials and Methods

### 2.1. Animals

Sprague Dawley rats (10 weeks old, 280–300 g, DBL, Seoul, Republic of Korea) were used and were kept under controlled conditions: 50% humidity at 24 ± 1 °C on a 12 h light–dark cycle. Standard pellet was purchased from Samtako (Osan, Republic of Korea) and rats had free access to the food and water. Animals were euthanized with Co2 inhalation and cervical dislocation. Ethical approval for the animal experimentation was obtained from the Kangwon National University Animal Ethics Committee (IACUC approval number KW-190226-1), and experiments were conducted following guidelines.

**Figure 1:**
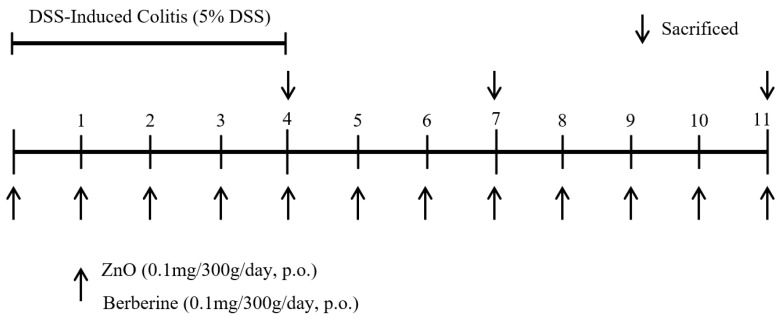
Experimental schedules for evaluating the effect of ZnO or berberine on the DSS-induced colitis. For colitis, male SD rats were administered 5% DSS in the drinking water for 4 days, then regular water for 7 days. ZnO (0.1 mg/300 g) or berberine (0.1 mg/300 g) was given daily by oral gavage. Animals were sacrificed at the indicated time points (4, 7 and 11 days).

Rats were randomly divided into four groups, with 15 animals in each group. The control group (Group 1) received sterile saline and standard food. In the 5% DSS group (Group 2), rats were allowed to freely drink a 5% DSS solution (MP Biomedicals, Santa Ana, CA, USA) for 4 days, followed by sterile saline for 7 days. To compare the anti-inflammatory effects of zinc oxide (ZnO) and berberine in DSS-induced colitis, ZnO (Group 3) and berberine (Group 4) were orally administered to the rats once a day (0.1 mg/300 g) while providing 5% DSS in the sterile saline. After discontinuation of DSS intake, treatment with ZnO and berberine continued for the following 7 days. We used a lower amount of ZnO feeding (150 ppm) than the European guidelines [23,24]. The concentration of DSS was determined by preliminary experiments, and the berberine dose was determined by our previous study [25]. To evaluate the sequential effects of berberine and ZnO, rats were sacrificed on days 4, 7, and 11 (Figure 1) according to a previous experimental schedule [26].

### 2.2. Body Weight, Fecal Water Contents, Disease Activity Index (DAI), and Colon Length

Body weight was measured every day and stool samples were observed for changes in fecal water content. Feces were collected immediately after defecation. Samples were sprayed with 5% HCl, weighed, and then dried in an oven at 70 °C for 72 h. The difference in weight before and after drying was used to calculate fecal water content.

**Figure 2:**
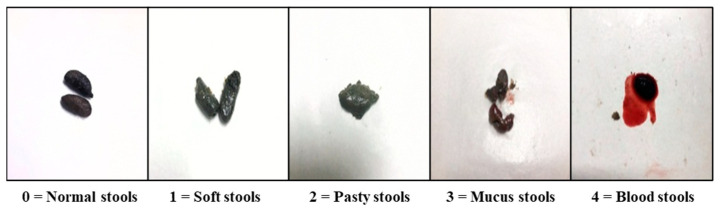
Rectal bleeding scores in rats with DSS-induced colitis. Scoring criteria accounted for the changes in stool consistency and humidity as follows: 0 = normal stool consistency and dryness, 1 = wet stools, 2 = pasty stools, 3 = with mucus and slightly bloody stools, 4 = blood stools.

The DAI was determined by combining scores from three categories: body weight loss, stool consistency, and the presence of blood in the stool [27,28]. Rectal bleeding are shown in Figure 2 [29]. Colon length changes were determined by the length from the proximal colon (excluding the cecum) to the distal colon [30].

### 2.3. Blood Collection and Analysis

Collected blood was incubated at room temperature for 30 min and then centrifuged at 1000 rpm for 10 min to obtain serum. The samples were stored at −80 °C until cytokine measurements were taken. Cytokine levels (TNF-α, IL-1β, and IL-6) were measured using the ELISA KOMA Cytokine ELISA kit (Komabiotech, Seoul, Republic of Korea) following the manufacture’s manual. Results were read at 450 nm using a microtiter plate reader (Molecular Devices, CA, USA). For albumin, ALP, AST, and ALP analyses, serum samples were sent to the Green Cross LABCELL (Gyeonggi-do, Republic of Korea).

### 2.4. Histology

The cecum was excised from each rat and placed in a cassette in a Swiss roll shape. Cecum and liver samples were fixed in 10% neutral-buffered formalin (NBF) and H&E staining was conducted.

### 2.5. Statistical Analysis

A two-way ANOVA test was used for statistical analysis using GraphPad Prism 5 (GraphPad Software, Boston, MA, USA) and *p*-values and fixed coefficients of the analysis from variance are indicated.

## 3. Results

### 3.1. Disease Activity

**Figure 3:**
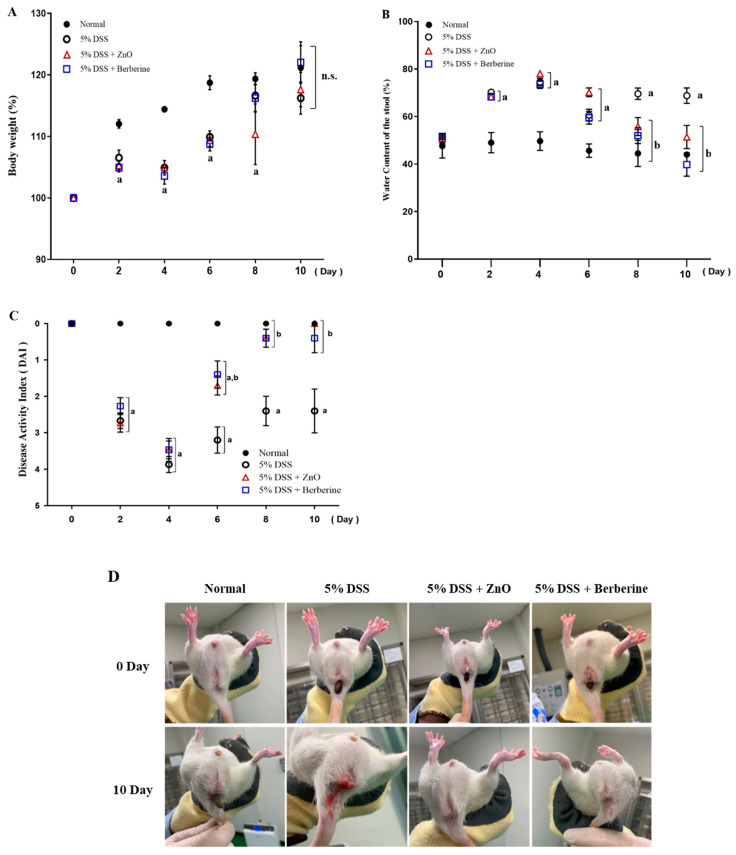
ZnO and berberine mitigated the clinical symptoms in DSS-induced colitis in rats. (**A**) Body weight was shown as a mean value of daily from 1 to 10. (**B**) Water content of the stool. (**C**) Disease activity index (DAI) scores. (**D**) Photographs of viable blood observation in rats. Data are expressed as mean ± SEM (*n* = 5). ^a^ *p* < 0.05 vs. normal group. ^b^ *p* < 0.05 vs. 5% DSS group. ^n.s.^ means that there is no statistical significance with each other.

Treatment of rats with 5% DSS significantly decreased body weight. However, treatment with ZnO (Group 3) and berberine (Group 4) rescued the weight loss caused by DSS treatment. Moreover, the mean weight of the berberine-treated group was similar to that of the normal group (Group 1) at day 8, although a significant change was not observed between Group 4 and Group 2. At day 10, there was no significant difference in weight gain between all groups (Figure 3A). DSS-induced colitis significantly increased the fecal water content above the levels in the normal group and both ZnO and berberine treatment reduced the fecal water at day 8 (Figure 3B). 

The DAI was determined by combining the decrease in body weight, severity of diarrhea, and degree of rectal bleeding. The measurement methods for the DAI are presented in Table 1, with an example of the assessment of the rectal bleeding index illustrated in Figure 2.

Rectal bleeding around the anus was observed in rats (Figure 3D). At days 2 and 4, disease activity was exacerbated in all DSS-treated groups. However, both ZnO and berberine treatment ameliorated disease activity and reduced the DAI index after day 6 (Figure 3C).

**Figure 4:**
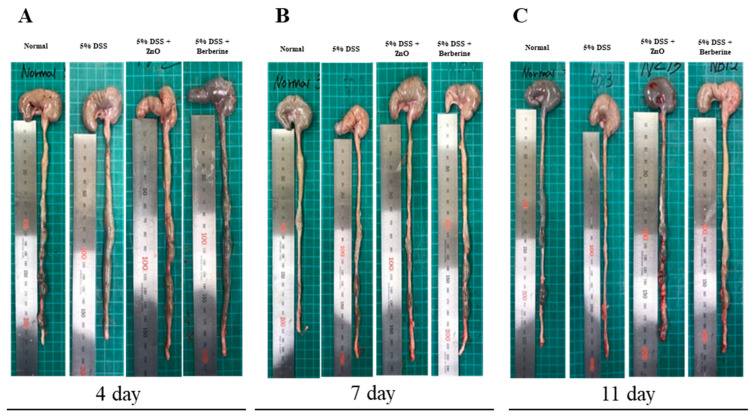
Effects of ZnO or berberine on colon length in rats with DSS-induced colitis. Morphology of colonic tissue at day 4 (**A**), day 7 (**B**), and day 11 (**C**) during DSS-induced colitis.

To evaluate additional protective effects of berberine, we assessed colon length during the DSS-induced colitis. On day 4, the average length was 22 ± 0.1 cm in the normal group, while 5% DSS treatment significantly decreased the average length to 16 ± 1.3 cm. ZnO and berberine treatments resulted in lengths of 18.5 ± 0.9 cm and 19.7 ± 0.6, respectively. On day 7, the 5% DSS group (Group 2) was still shorter (19.5 ± 0.9 cm) than the normal group (Group 1). Neither ZnO nor berberine treatment significantly altered colon length compared with the 5% DSS group. On day 11, the colon mean length in the normal group was 23.8 ± 0.8 cm, while in the 5% DSS group, it was 20.2 ± 0.6 cm. Colon lengths were significantly longer in the ZnO-treated (21.1 ± 0.3 cm) and berberine-treated (21.5 ± 0.5 cm) groups than in the 5% DSS group (Table 2 and Figure 4). These results indicate that berberine treatment has a protective effect similar to ZnO against symptoms of DSS-induced colitis.

### 3.2. Regulatory Action of Berberine in DSS-Induced Colitis

**Figure 5:**
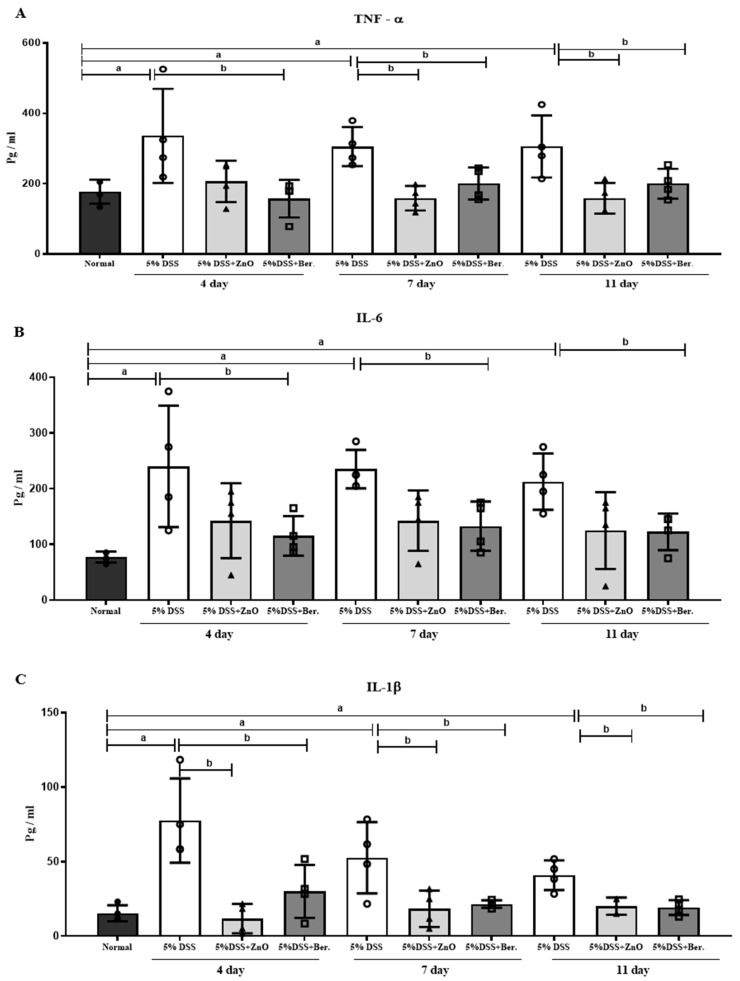
Effects of ZnO or berberine on serum levels of inflammatory cytokines in rats with DSS-induced colitis. (**A**) TNF-α, (**B**) IL-6, and (**C**) IL-1β levels were examined by ELISA. Data are expressed as mean ± S.E.M (*n* = 5). ^a^ *p* < 0.05 compared with normal group. ^b^ *p* < 0.05 compared with 5% DSS group.

To examine the anti-inflammatory effects of ZnO and berberine during intestinal inflammation, levels of major cytokines, such as TNF-α, IL-6, and IL-1β, were measured in blood serum. As expected, cytokine levels were significantly increased in the 5% DSS group on days 4, 7, and 11 (Figure 5). However, these increases were not observed after the administration of ZnO or berberine, and there was no significant difference between cytokine levels in the 5% DSS + ZnO group and the 5% DSS + berberine group.

**Figure 6:**
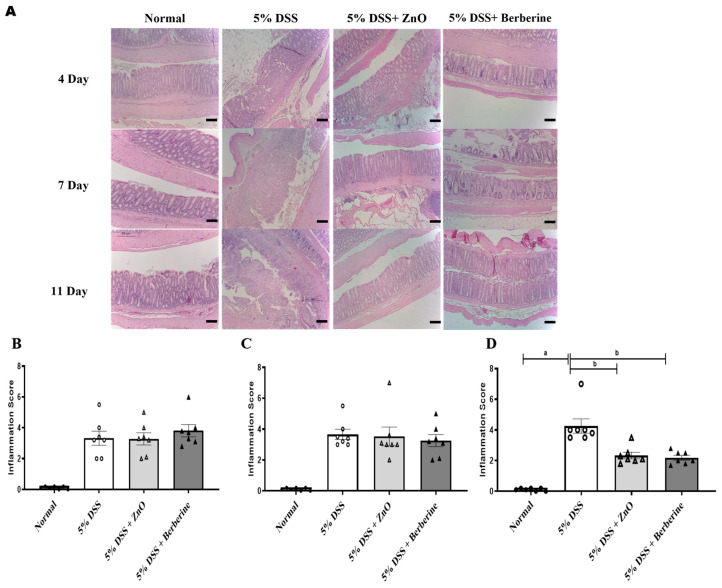
Effect of ZnO and berberine on colonic histopathological changes in rats with DSS-induced colitis. (**A**) Representative H&E staining image of colonic sections from different treatment. Magnification ×200. Bar, 200 µm. (**B**) Inflammation scores at 4 days, (**C**) at 7 days, and (**D**) at 11 days. Data are expressed as mean ± S.E.M (*n* = 5). ^a^ *p* < 0.05 compared with normal group. ^b^ *p* < 0.05 compared with 5% DSS group.

Next, we evaluated the possible action of berberine on the histopathology of the colon. Relative to the normal group, the 5% DSS group displayed destruction of crypt structures, changes in crypt length, and infiltration of inflammatory cells in all colonic tissues on days 4, 7, and 11 (Figure 6A). These changes were consistently observed in the colon of the 5% DSS + ZnO group and the 5% DSS + berberine group on days 4 and 7 (Figure 6B,C). However, on day 11, restoration of crypt structure and a reduction in the infiltration of inflammatory cells were observed in the colon tissues of the 5% DSS + ZnO group and the 5% DSS + berberine group (Figure 6D). These data demonstrate that berberine has similar anti-inflammatory actions to ZnO in DSS-induced colitis.

### 3.3. Regulatory Action of Berberine in DSS-Induced Colitis Albumin, AST, ALT, and ALP Levels in Serum

**Figure 7:**
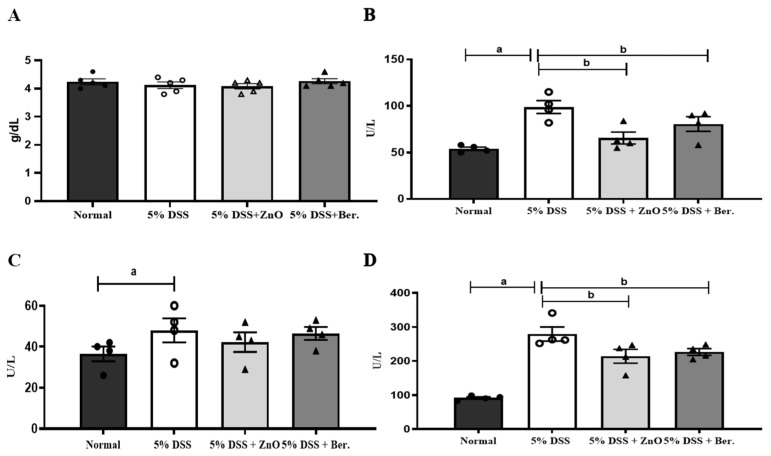
Effects of ZnO or berberine on serum levels of albumin, AST, ALT, and ALP in rats with DSS-induced colitis. (**A**) Albumin, (**B**) AST, (**C**) ALT, and (**D**) ALP levels were measured in the rat serum. Data are expressed as mean ± S.E.M (*n* = 5). ^a^ *p* < 0.05 compared with normal group. ^b^ *p* < 0.05 compared with 5% DSS group.

**Figure 8:**
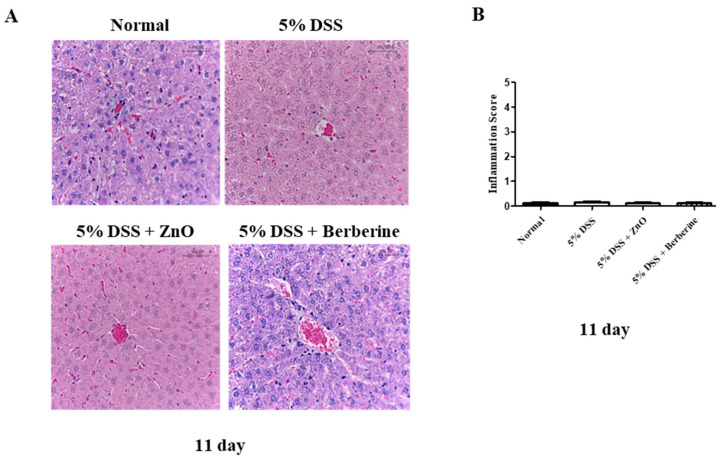
Effect of ZnO or berberine on hepatic histopathological changes in rats with DSS-induced colitis. (**A**) Representative H&E staining image of hepatic sections from different treatment. Magnification ×200. Bar, 50 µm. (**B**) Inflammation scores at day 11. Data are expressed as mean ± S.E.M (*n* = 5).

We also investigated the potential impact of ZnO and berberine hepatic damage. Liver function was estimated by determining the levels of albumin, AST, ALT, and ALP. No changes were observed in albumin concentration in any of the experimental groups (Figure 7A). On day 11, the levels of AST and ALP were increased in the 5% DSS group compared with the normal group (Figure 7B,D). However, these levels were significantly lower in the 5% DSS + ZnO and the 5% DSS + berberine groups. Notably, the ALT level was higher in the 5% DSS group than the normal group, whereas it was not significantly changed or different from the control in the 5% DSS + ZnO and the 5% DSS + berberine groups (Figure 7C). Additionally, although changes in hepatic enzymes were observed in the 5% DSS groups, there were no histological changes in liver tissues in any group (Figure 8). A partial suppression of liver enzymes was produced by berberine during colitis, but the 5% DSS treatment did not produce any liver histopathology.

## 4. Discussion

The primary purpose of treating livestock with ZnO is to prevent diarrhea by ameliorating colitis [31]. The amount of ZnO in piglet feed is highly concentrated, which is potentially excreted in the feces, thereby contributes to environmental contamination. [5,10]. Several studies have attempted to minimize adverse effects by using nanoparticle ZnO to increase intestinal absorption. However, there are few studies comparing the action of ZnO with berberine, which could potentially replace ZnO [32]. In order to reduce the levels of environmental pollution produced by livestock farming, it is necessary to find alternative substances to ZnO. 

The DSS colitis model represents major clinical symptoms of in animal models, such as intestinal epithelium necrosis and bloody diarrhea [33]. This study using a rat model of DSS-induced colitis suggests that berberine could be a valid substitute for ZnO in livestock feed. Berberine displayed anti-inflammatory effects in DSS-induced colitis, which caused damage to the colonic epithelial barrier, exposure of the mucosa to bacteria, infiltration of inflammatory cells, epithelial damage, and ulcer formation [34,35]. DSS administration increases the level of effector cytokines, such as TNF-α, IL-6, and IL-1β in the blood, which induces inflammation in colonic epithelial cells and alters ion permeability [36]. This imbalance in ion transport leads to severe diarrhea and rapid weight loss.

In this study, DSS-treated rats displayed high serum levels of TNF-α, IL-6, and IL-1β. In contrast, DSS-treated rats administered berberine displayed lower levels of these inflammatory indexes, consistent with a previous study that observed an inhibitory action of berberine on inflammatory cytokines in models of DSS-induced colitis [37]. It was also reported that berberine effectively inhibits the activation of the IL-6/STAT3/NF-κB pathway in models of DSS-induced colitis, offering a potential treatment for ulcerative colitis. Although we did not investigate STAT3/NF-κB activation, the serum level of IL-6 significantly decreased by berberine treatment. The action of berberine and ZnO potentially depend on IL-6-associated STAT3/NF-κB pathway regulation [38].

Furthermore, we examined the levels of other inflammatory cytokines, including TNF-α and IL-1β, which were lowered by berberine treatment. This regulatory effect was similar to that of ZnO treatment. Notably, however, only a few studies have examined the protective effects of ZnO in the DSS-induced colitis model. For example, a previous study reported that ZnO nanoparticles reduced the elevated concentrations of IL-1β and TNF-α in a model of DSS-induced colitis [28]. In a piglet model, the regulation of effector cytokines such as IL-1, IL-6, and TNF-α in the colon is closely related to animal growth and inhibition of diarrhea [39]. In line with this, our study observed the suppression of these inflammatory molecules using berberine and ZnO, which indicates the possibility of berberine as a ZnO alternative. 

DSS administration induced histological changes in colon tissues at days 4, 7, and 11. However, berberine and ZnO treatments significantly reduced inflammatory responses at day 11. The delayed appearance of the anti-inflammatory effects of berberine and ZnO may be due to the initial suppression of inflammation-inducing cytokines in the blood, leading to subsequent tissue recovery. This trend was consistent with the DAI, and improved tissue inflammation seemed to be correlated with physiological indicators. 

In DSS-treated rats administered ZnO or berberine, the water content in the feces gradually recovered to levels not significantly different to those in the normal group. Colon length was not significantly shortened, indicating rapid recovery. These findings suggest that ZnO and berberine have a similar capacity to improve the physiological functions of colon tissues, as well as reduce inflammation. Salmonella Typhimurium infection disrupts gut tight junctions, increasing the permeability of intestinal epithelium. Pathogenic antigens then invade the lamina propria, leading to intestinal inflammation [40]. Although we have not investigated the effect of ZnO and berberine on the integrity of gut epithelium, they ameliorated intestinal histopathology (Figure 8). The treatments are believed to have enhanced colon function, leading to improvements in the DAI and increased colon length (Figure 3C and Figure 4, Table 2). We performed a pilot study to determine the optimal condition using 2%, 3%, and 5% DSS. DAI and colon length was significantly different at each concentration after 9 days post-administration. Therefore, we concluded that 5% DSS is valid for colitis induction, independent of drug interference. Additionally, we observed that body weight recovered after DSS treatment, with variations observed within the standard deviation. This indicates that each animal consumed a comparable amount of food.

The additive in piglet food should be non-toxic to the liver [41]. We also investigated whether berberine induced toxicity in the liver. Albumin is an indicator of liver synthesis, as it is exclusively synthesized in the liver and constitutes a major protein [42]. However, in this study, no significant differences in albumin levels were observed across all the experimental groups. AST and ALT levels were increased in the 5% DSS group, but there were no significant differences in AST and ALT levels between the ZnO and berberine groups, which were also similar to the normal group. Alkaline phosphatase (ALP) is an enzyme involved in the transport of metabolites through the cell membrane [42]. In this study, the ALP level was significantly higher in the 5% DSS-treated group than in the normal group. In contrast, the 5% DSS + ZnO and 5% DSS + berberine groups displayed lower ALP levels than the 5% DSS group. In addition, ZnO and berberine did not induce hepatic damage, based on the levels of AST, ALT, ALP, and albumin. Lastly, histological analysis indicated that 5% DSS treatment and ZnO and berberine administration did not induce liver damage. The results indicate that ZnO and berberine treatments are non-toxic to the liver, supporting the safety of berberine as a feed additive. 

## 5. Conclusions

In summary, our study revealed that ZnO and berberine exhibit anti-inflammatory effects in a rat model of DSS-induced colitis. Both treatments limited the production of inflammatory cytokines, which improved the physiological function of the intestine, without liver toxicity. The protective effects of ZnO and berberine were observed at the same dosage and the dose of berberine is economically viable for livestock use, while the ZnO dosage (150 ppm) is lower than the EU recommendation [23,24]. In light of our findings, further studies should be performed with different dosages and in different disease models, involving piglets, but the current findings indicate that berberine could be used as a substitute for ZnO in livestock feed.

## Figures and Tables

**Table 1:** Clinical signs of DSS animals.

Score	Body Weight Change (%)	Diarrhea	Rectal Bleeding
0	None	None	Normal stools
1	1~5	Mild	Wet stools
2	5~10		Pasty stools
3	11~15		With mucus stools
4	15~20	Severe watery	Blood stools

**Table 2:** Colon length of DSS animals.

	4 Day	7 Day	11 Day
Normal	22 ± 0.1	22.7 ± 0.3	23.8 ± 0.9
5% DSS	16 ± 1.3	19.5 ± 0.9	20.2 ± 0.6
5% DSS + ZnO	18.5 ± 0.9	20.5 ± 0.8	21.1 ± 0.3
5% DSS + Berberine	19.7 ± 0.6	20.9 ± 0.6	21.5 ± 0.5

The table shows the data of colon length. Data are expressed as mean ± S.E.M (*n* = 5).

## Data Availability

Data are contained within the article.
